# Seroprevalence and Determinants of ToRCH Pathogens in Pregnant Women in the Sub-Himalayan Region

**DOI:** 10.7759/cureus.21946

**Published:** 2022-02-05

**Authors:** Sangeeta Deka, Deepjyoti Kalita, Manisha Paul, Gaurav Badoni, Yogendra P Mathuria

**Affiliations:** 1 Microbiology, All India Institute of Medical Sciences, Rishikesh, Rishikesh, IND; 2 Microbiology, Fakhruddin Ali Ahmed Medical College, Barpeta, IND

**Keywords:** torch, india, antenatal screening, pregnant women, herpes simplex virus, cytomegalovirus, rubella virus, toxoplasma gondii

## Abstract

Introduction

*Toxoplasma gondii *(TG), rubella virus (RV), cytomegalovirus (CMV), and herpes simplex virus type 1 and 2 (HSV 1 and 2) cause mild maternal morbidity but have serious fetal consequences. The prevalence of these infections varies widely by country and population subgroup, and the paucity of data from the hilly state of Uttarakhand prompted us to undertake this study on their seroprevalence and association with potential risk factors.

Methods

Serum samples received from pregnant women attending the antenatal clinic of All India Institute of Medical Sciences, Rishikesh, between January 2016 to December 2019 were tested for TG-, RV-, CMV, and HSV-specific IgM and IgG by capture enzyme-linked immunoassay (ELISA). The data were then analyzed to determine the seroprevalence of the major ToRCH infections (toxoplasmosis, other (syphilis, varicella-zoster, parvovirus B19), rubella, cytomegalovirus, and herpes), and Fisher’s exact test was applied to check association with potential risk factors.

Results

Out of 165 pregnant women who were screened for the four major ToRCH pathogens, overall seroprevalence was 41.2% for TG (IgM=13.3%; IgG=38.2%), 80.0% for RV (IgM=3.0%; IgG=80.0%), 61.8% for CMV (IgM=1.8%; IgG=61.8%), and 42.4% for HSV (IgM=4.3%; IgG=40.6). TG was significantly associated with increasing maternal age (p-value=0.007). The seropositivity of RV was maximum in the drier and windy months of January-March (p-value=0.004), while that of TG in the warmer months of April-June (p-value=0.03). HSV prevalence was comparatively more common in Muslim women (p-value=0.05). Women presenting with bad obstetric history (BOH) and multiparous women were at higher risk for TG-RV-HSV and TG-RV-CMV, respectively.

Conclusion

Considering the high prevalence and risk of ToRCH infections in this region, we suggest disease-specific screening based on maternal history. Recognition of the burden of ToRCH infections in pregnant women is vital in clinicians’ decisions and implementing control measures.

## Introduction

Commonly accepted thoughts about *Toxoplasma gondii *(TG), rubella virus (RV), cytomegalovirus (CMV), and herpes simplex virus type 1 and 2 (HSV 1 and 2) is that they commonly cause very mild illness in immunocompetent adults, but when the infection is acquired during pregnancy, they can cause serious complications in the fetus and the newborn. These complications range from intrauterine growth restriction (IUGR), congenital malformations (mild to long-term sequelae) to fetal death depending upon the gestational age during transplacental infection [1-3]. They are transmitted prenatally (transplacental transmission), perinatally (exposure to blood and vaginal secretion during birth), or, rarely, postnatally (breastfeeding). Nearly 3% of all congenital anomalies are caused due to prenatal infections by these microbes [1,4]. Pregnant women are more susceptible to certain infectious diseases due to a state of selective immune tolerance, immunosuppression, and immunomodulation for physiological adaptation. Additionally, the immature and developing immune system of the fetus is unable to resist the proliferation of infectious agents that cross the placental barrier [3,5]. 

Due to the mostly asymptomatic or mild clinical course of these four diseases, diagnosis during pregnancy is often missed [4,6]. So, the most effective way to control birth defects due to prenatal infection by these four microorganisms is preventive antenatal screening and counseling, leading to early diagnosis [6,7]. A screen for toxoplasmosis, other (syphilis, varicella-zoster, parvovirus B19), rubella, cytomegalovirus, and herpes (ToRCH screen) is a panel of serological tests used to screen pregnant women for these infections. T, R, C, and H in the acronym ToRCH panel stand for TG, RV, CMV, and HSV, respectively.

TG, an apicomplexan parasite, is mainly caused by ingestion of *T. gondii *oocyst present on raw and unwashed vegetables or undercooked meat containing tissue cysts [7]. During pregnancy, primary TG infection or reactivation may cause congenital toxoplasmosis with serious fetal outcomes like hydrocephalus, retinitis pigmentosa, or even death in utero. Rubella or German measles is an airborne viral infection characterized by fever, upper respiratory infection, skin rash, lymphadenopathy, and joint pain. Congenital rubella syndrome (CRS) can include spontaneous abortion, stillbirth, IUGR (in up to 60% of newborns), hepatosplenomegaly, thrombocytopenia, and purple rash [1]. The infected newborn may have vision and/or hearing impairment, heart defects, and calcium deposits in the brain. CMV, regarded as human herpesvirus (HHV) type-5, usually causes asymptomatic infections in immunocompetent adults. But infected infants may present with microcephaly, hepatosplenomegaly, hepatitis, hemolytic anemia, and/or other abnormalities. Neonatal herpes, caused by HSV-1 and 2, is usually acquired from infected mothers with genital lesions during delivery. It may vary from cutaneous blisters, conjunctivitis, hepatitis, central nervous system manifestations.

Serological tests are the mainstay for diagnosing ToRCH infections [8]. The most commonly used test is enzyme-linked immunosorbent assay (ELISA) for detecting IgM and IgG antibodies against these pathogens. Other assays, like automated chemiluminescent immunoassay (quantitative), indirect immunofluorescence assay, and lateral flow chromatographic immunoassay (both qualitative), for detecting virus-specific immunoglobulins (IgM and IgG) can also be done [8,9,10]. IgG avidity test plays a vital role in differentiating patients with acute infection from those with chronic infection. Molecular assays like polymerase chain reaction (PCR) have high sensitivity and specificity but are applicable only for molecular typing and not for routine screening in resource-scant settings.

As there is a paucity of studies on seroprevalence of ToRCH agents in pregnant women from the sub-Himalayan state of Uttarakhand, this study aimed to assess the seroprevalence of TG, RV, CMV, and HSV among pregnant women visiting All India Institute of Medical Sciences, Rishikesh, for an antenatal check-up and to find its correlation with socio-demographic characteristics and bad obstetrics history (BOH).

## Materials and methods

Study area

**Figure 1:**
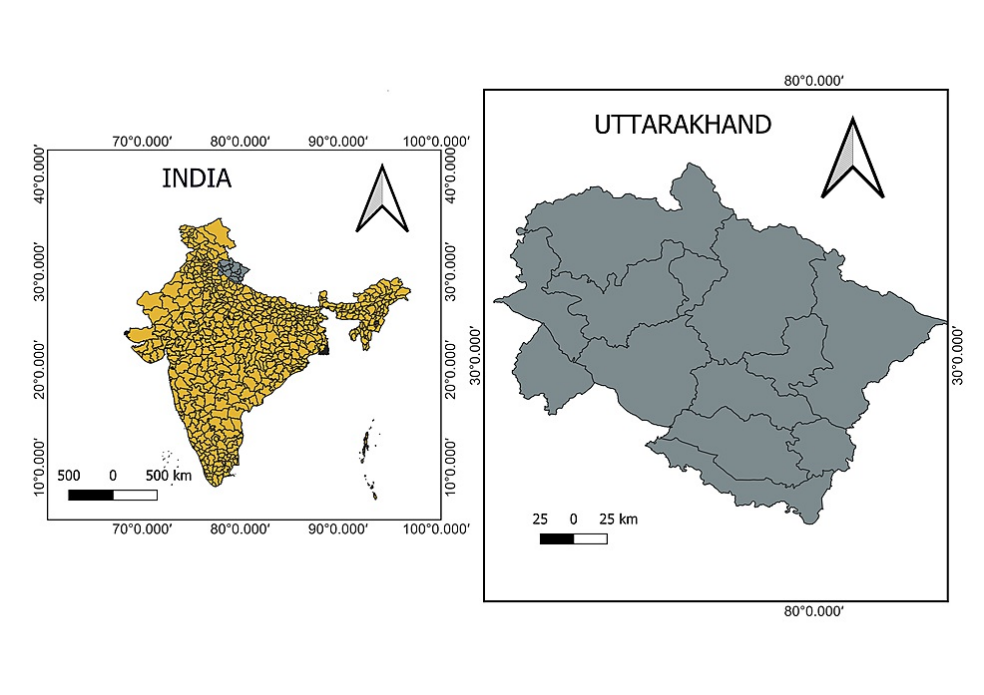
Map of the study area

This cross-sectional study was conducted in the microbiology department of the All India Institute of Medical Sciences, Rishikesh, in Uttarakhand, India, between January 2016 and December 2019. It is a tertiary care teaching hospital offering healthcare services to patients from the sub-Himalayan state of Uttarakhand and adjoining areas. This area comprises both river valley plains and hilly terrains. Map of the area covered in the study, created using the QGIS application, is depicted in Figure 1.

Inclusion and exclusion criteria

All pregnant women attending the antenatal and gynecological outpatient department (OPD) of our institute for routine antenatal check-ups for four years (2016-2019) were considered for the study. Out of these, subjects who underwent screening for ToRCH pathogens, either as a routine antenatal screening process or had BOH or signs/symptoms compatible with those of ToRCH infections, were enrolled in the study. BOH implies previous unfavorable fetal outcomes in terms of two or more consecutive spontaneous abortions, history of fetal death/s, IUGR, early neonatal death, and/or congenital anomalies. Data used in the study were collected primarily from selected subjects or their attendants (carrying the samples). In case of any information gap, we referred to laboratory register/medical records maintained in the department or hospital information system (HIS). Cases with incomplete data were excluded from the study.

Laboratory methods

Blood samples were collected from the cases aseptically in plain vials. After half an hour, they were centrifuged and serum was separated. Aliquoted sera were stored at a -20 degree centigrade deep freezer until further processed. IgG and IgM levels for TG, RV, CMV, and HSV were measured using Calbiotech IgG and IgM ELISA kits (Calbiotech R5EC 96 well ELISA, El Cajon, USA) individually for each organism. ELISA was performed on automated Euroimmune Analyzer 1 (Walkaway automated seven plate ELISA reader; Euroimmun, Lübeck, Germany), and the optical density (OD) was read at 450nm absorbance. All the ELISA kits had 99% Kappa agreement with reference ELISA method according to manufacturer’s kit insert. The interpretation of test results was based on the antibody index (AI) calculated by dividing the OD value of each sample by cut-off value (calibrator OD multiplied by calibrator factor). An AI of >1.1 was interpreted as positive and <0.9 was regarded as negative result. An AI of 0.9-1.1 was considered equivocal.

Statistical analysis

Data were entered into an Excel spreadsheet (Microsoft® Corp., Redmond, USA) separately by two researchers and checked for errors. The standard software package of SPSS Statistics for Windows, version 23.0 (IBM Corp., Armonk, NY), was used for all statistical analyses. Mean and standard deviation was calculated for overall IgM and IgG levels against TG, RV, CMV, and HSV. The differences in the mean of dependent variable (IgM and IgG levels against pathogen) for each set of independent variables (age groups, year, months, religion, parity and BOH) were also checked. Associations of ToRCH infections with the independent variables were calculated using Fisher’s exact test. A two-tailed p-value of <0.5 was considered significant.

Ethical declaration

The present study protocol was reviewed and approved by the institutional review board of our institute (AIIMS/IEC/20/835 Date: 12/12/2020). Patient confidentiality was maintained by excluding their name and registration number while compiling the data and giving a specific identifier number.

## Results

Patient characteristics

Altogether 4053 pregnant women attended the antenatal and obstetrics and gynecology OPD for routine antenatal check-ups between January 2016 and December 2019. Among them, 196 (4.8%) women, with or without any clinical presentations and/or BOH, were screened for the four major ToRCH infections. Thirty-one women were excluded from the study due to the unavailability of required data, and finally, the remaining 165 women were further analyzed in the study. The mean age of studied subjects was 27.6 ± 5.9 years. Table 1 shows the distribution of the study population. The majority of the cases belonged to 21-25 years (35.2%), followed by 26-30 years. The number of cases gradually increased from 14.5% in 2016 to 34.5% in2019. The cases were equally distributed over the year, but a spike in cases was noticed in April and September. Most pregnant women belonged to the Hindu religion (78.8%), while 17.6 practiced Islam. About 27.3% of women were pregnant with their first viable child, while 29.1% of the women had presented with BOH.

**Table 1:** Distribution of study participants according to socio-demographic characteristics N= 165 * For the age category, the lower limit is the completed given age (in years) and the higher limit is completed given age (in years) up to one day less than the succeeding year. # Women that have had more than one pregnancy resulting in viable offspring. BOH, bad obstetrics history

Characteristics		No. of cases, n	Percentage %
Maternal age groups *	<20	15	9.1
	21-25	58	35.2
	26-30	52	31.5
	>31	40	24.2
Year	2016	24	14.5
	2017	33	20.0
	2018	51	30.9
	2019	57	34.5
Time of year	Jan-Mar	36	21.8
	Apr-Jun	46	27.9
	Jul-Sep	45	27.3
	Oct-Dec	38	23.0
Religion	Hindu	130	78.8
	Muslim	29	17.6
	Others	6	3.6
Parity	Primipara	43	26.1
	Multipara #	122	73.9
BOH	Yes	48	29.1
	No	117	70.9

Sero-prevalence of the ToRCH pathogens

The seroprevalence of the four main ToRCH pathogens is displayed in Table 2. The overall seropositivity of TG was 41.2%. Maximum total seropositivity was found in RV (80.0%), followed by CMV (61.8`%) and HSV (42.4%). While IgG against TG, RV, CMV, and HSV was elevated in 38.2%, 80.0%, 61.8%, and 40.6%, respectively, IgM was raised only in 13.3%, 3%, 1.8%, and 4.3%, respectively. Both IgM and IgG were raised against TG, RV, CMV and HSV in 10.9%, 3% , 1.3%, and 2.4% cases, respectively.

**Table 2:** Serological status of IgG and IgM antibodies with mean and standard deviation against Toxoplasma gondii, rubella, cytomegalovirus, and herpes simplex virus type 1 and 2 infections in pregnant women AI: antibody index; EV: Equivocal

		Toxoplasma gondii	Rubella virus	Cytomegalovirus	Herpes virus
IgM	Positive n,(%)	22 (13.3)	5 (3.0)	3 (1.8)	7 (4.3)
	EV n,(%)	10 (6.1)	1 (0.6)	0	2 (1.2)
	Negative n,(%)	133 (80.6)	159 (96.4)	162 (98.2)	156 (94.65)
	Mean AI	0.74	0.42	0.29	0.33
	SD	+/- 1.2	+/- 1.4	+/- 0.4	+/- 0.27
IgG	Positive n,(%)	63 (38.2)	132 (80.0)	102 (61.8)	67 (40.6)
	EV n(,%)	4 (2.4)	2 (1.2)	3 (1.8)	3 (1.8)
	Negative n,(%)	98 (69.4)	31 (18.8)	60 (36.4)	95 (57.6)
	Mean AI	41.9	81.2	5.63	3.34
	SD	+/- 124.6	+/- 267.7	+/- 14.4	+/- 14.9
Both IgG & IgM	Positive n,(%)	18 (10.9)	5 (3.0)	3 (1.8)	4 (2.4)
Total	Positive n,(%)	68 (41.2)	132 (80.0)	102 (61.8)	70 (42.4)
	EV n,(%)	7 (4.3)	2 (1.2)	3 (1.8)	3 (1.8)
	Negative n,(%)	90 (54.5)	33 (20.0)	63 (38.2)	92 (55.8)

Associated factors

**Figure 2:**
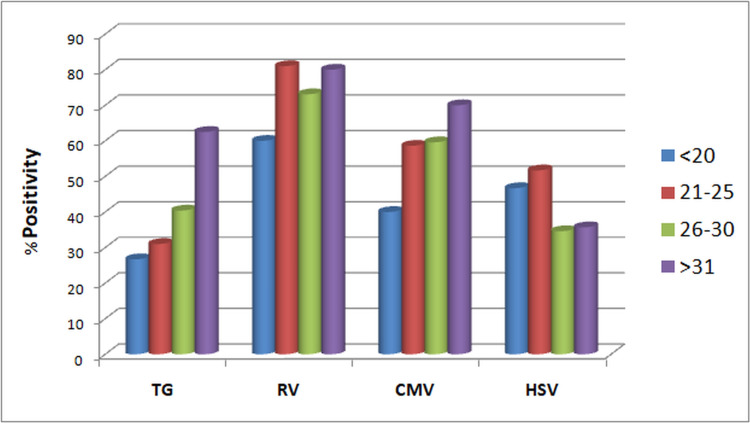
Prevalence of Toxoplasma gondii (TG), rubella virus (RV), cytomegalovirus (CMV), and herpes simplex virus (HSV) infections in different age groups of pregnant women

**Figure 3:**
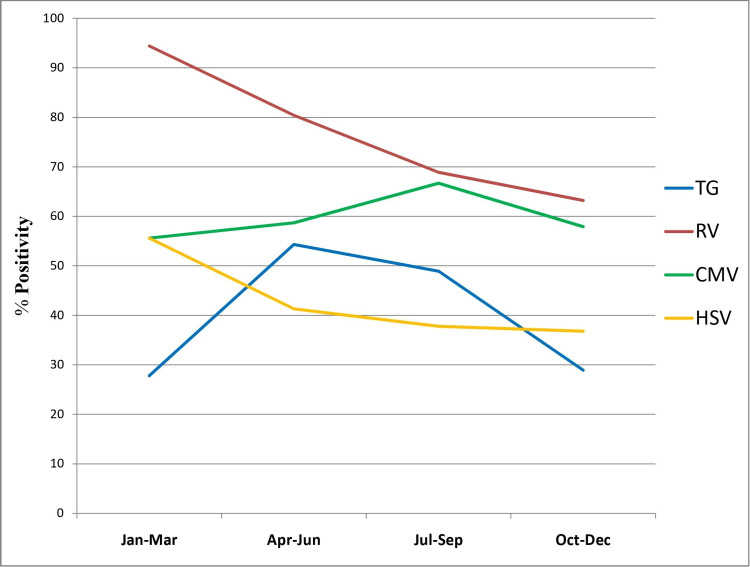
Trend of seropositivity of ToRCH infections in the different months of a year TG, Toxoplasma gondii; RV, rubella virus; CMV, cytomegalovirus; HSV, herpes simplex virus

**Figure 4:**
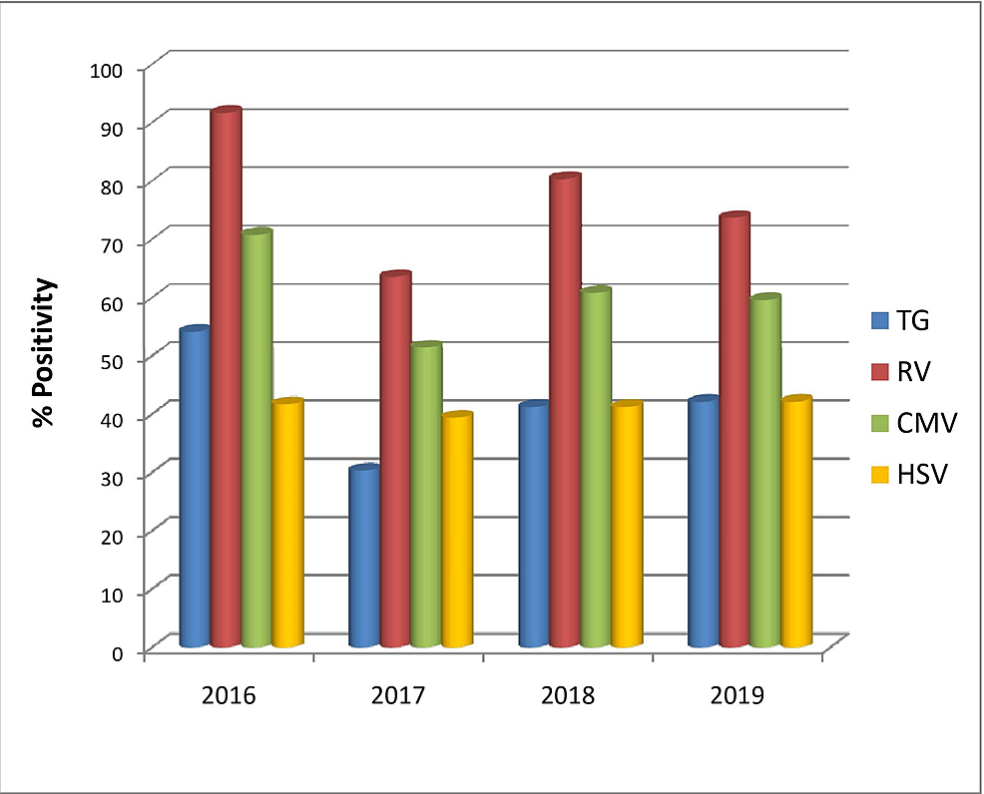
Trend of seropositivity of ToRCH infections in the four years of the study period TG, Toxoplasma gondii; RV, rubella virus; CMV, cytomegalovirus; HSV, herpes simplex virus

When demographic factors were taken into consideration, TG seroprevalence significantly increased with increasing age groups (p-value= 0.007), reaching the maximum rate at >31 years (62.5%) (Figure 2). The rate of TG and RV seroprevalence was higher during April-June and January-March, respectively (TG p-value=0.031, RV p-value=0.004) (Figure 3). However, no significant changes in prevalence were noticed in the four years (Figure 4). HSV was more prevalent in Muslim women (p-value=0.050) (Table 3). Multiparous women had a significantly higher risk of having anti-TG, -RV, and -CMV antibodies, whereas women with BOH were significantly associated with TG, RV, and HSV (Table 3). Table 4 describes the difference in means and standard deviations among the different categories.

**Table 3:** Results of Fisher’s exact test to check the association of ToRCH infections with probable risk factors BOH: bad obstetrics history; Pos: Positive; Neg: Negative; *sig.: significance (2-sided p-value) by Fisher's exact test

Characteristics	Toxoplasma gondii			Rubella virus					Cytomegalovirus			Herpes virus		
	Pos n, %	Neg n, %	Exact sig.*	Pos n, %		Neg n, %		Exact sig.*	Pos n, %	Neg n, %	Exact sig.*	Pos n, %	Neg n, %	Exact sig.*
Maternal Age Group														
<20	4 (26.7)	11 (73.3)	0.007	9 (60.0)		6 (40.0)		0.321	6 (40.0)	9 (60.0)	0.242	7 (46.7)	8 (53.8)	0.281
21-25	17 (29.4)	41 (70.7)		17 (81.0)		11 (19.0)			34 (58.6)	24 (41.4)		30 (51.7)	26 (48.3)	
26-30	21 (40.4)	31 (59.6)		38 (73.1)		14 (26.9)			31 (59.6)	21 (40.4)		18 (34.6)	34 (65.4)	
>31	25 (62.5)	15 (37.5)		32 (80.0)		8 (20.0)			28 (70.0)	12 (30.0)		15 (37.5)	25 (62.5)	
Year														
2016	13 (54.2)	11 (45.8)	0.344	22 (91.7)		2 (8.3)		0.082	17 (70.8)	7 (29.2)	0.543	10 (41.7)	14 (58.3)	0.984
2017	10 (30.3)	23 (69.7)		21 (63.6)		12 (36.4)			17 (51.5)	16 (48.5)		13 (39.4)	20 (60.6)	
2018	20 (39.2)	31 (60.8)		41 (80.4)		10 (19.6)			31 (60.8)	20 (39.2)		22 (43.1)	29 (56.9)	
2019	24 (42.1)	33 (57.9)		42 (73.7)		15 (26.3)			34 (59.6)	23 (40.4)		25 (43.9)	32 (56.1)	
Time of year														
Jan-Mar	10 (27.8)	26 (72.2)	0.031	34 (94.4)		2 (5.6)		0.004	20 (55.6)	16 (44.4)	0.748	20 (55.6)	16 (44.4)	0.341
Apr-Jun	25 (54.3)	21 (45.7)		37 (80.4)		9 (19.6)			27 (58.7)	19 (41.3)		19 (41.3)	27 (58.7)	
Jul-Sep	21 (46.7)	24 (53.8)		31 (68.9)		14 (31.1)			30 (66.7)	15 (33.3)		17 (37.8)	28 (62.2)	
Oct-Dec	11 (28.9)	27 (71.1)		24 (63.2)		14 (36.8)			22 (57.9)	16 (42.1)		14 (36.8)	24 (63.2)	
Religion														
Hindu	52 (40.0)	78 (60.0)	0.807	101 (77.7)		29 (22.3)		0.646	77 (59.2)	53 (40.8)	0.720	50 (38.5)	80 (61.5)	0.050
Muslim	13 (44.8)	16 (55.2)		21 (72.4)		8 (27.6)			19 (65.5)	10 (34.5)		18 (62.1)	11 (37.9)	
Others	2 (33.3)	4 (66.7)		4 (66.7)		2 (33.3)			3 (50.0)	3 (50.0)		2 (33.3)	4 (66.7)	
Parity														
Primipara	10 (23.3)	33 (76.7)	0.007	27 (62.8)	16 (37.2)		0.021		20 (46.5)	23 (53.5)	0.046	17 (39.5)	26 (60.5)	0.397
Multiparous	57 (46.7)	65 (53.3)		99 (81.9)	23 (18.9)				79 (64.8)	43 (35.2)		53 (43.4)	69 (56.6)	
BOH														
Present	41 (35.0)	76 (65.0)	0.026	84 (71.8)	33 (28.2)		0.043		66 (56.4)	51 (43.6)	0.097	43 (36.8)	74 (63.2)	0.035
Absent	26 (54.2)	22 (45.8)		42 (87.5)	6 (12.5)				33 (68.8)	15 (31.3)		27 (56.3)	21 (43.8)	

**Table 4:** Comparison of the mean and standard deviation of IgG and IgM levels of the ToRCH pathogens between the different groups of each variable BOH, bad obstetrics history; TG, Toxoplasma gondii; RV, rubella virus; CMV, cytomegalovirus; HSV, herpes simplex virus *For age category, the lower limit is the completed given age (in years) and the higher limit is completed given age (in years) up to one day less than the succeeding year ^Women who has had more than one pregnancy resulting in viable offspring

Characteristics		Mean + Standard Deviation							
		TG IgG	TG IgM	RV IgG	RV IgM	CMV IgG	CMV IgM	HSV IgG	HSV IgM
Maternal Age groups*	<20	0.6 +/- 0.8	0.5 +/- 0.4	68.5 +/- 257.7	0.2 +/- 0.2	2.7 +/- 4.5	0.2 +/- 0.2	1.8 +/- 2.6	0.2 +/- 0.2
	21-25	19.5 +/- 59.7	0.5 +/- 0.5	58.7 +/- 211.5	0.3 +/- 0.4	3.7 +/- 4.8	0.3 +/- 0.6	5.9 +/- 23.8	0.4 +/- 0.3
	26-30	51.9 +/- 143.6	0.7 +/- 1.1	95.7 +/- 313.2	0.7 +/- 2.1	4.5 +/- 12.9	0.3 +/- 0.3	1.1 +/- 1.6	0.3 +/- 0.2
	>31	72.3 +/- 167.6	1.3 +/- 1.8	99.9 +/- 287.2	0.3 +/- 0.2	10.9 +/- 23.8	0.3 +/- 0.2	3.1 +/- 9.1	0.2 +/- 0.1
Year	2016	56.2 +/- 146.6	0.9 +/- 1.5	182.6 +/- 436.2	0.6 +/- 1.8	4.9 +/- 6.3	0.3 +/- 0.2	1.2 +/- 1.7	0.4 +/- 0.3
	2017	34.1 +/- 107.6	0.6 +/- 0.9	42.4 +/- 146.9	0.3 +/- 0.2	3.0 +/- 4.2	0.2 +/- 0.2	4.6 +/- 16.7	0.3 +/- 0.3
	2018	43.5 +/- 129.0	0.8 +/- 1.2	75.7 +/- 252.2	0.5 +/- 1.3	9.7 +/- 24.3	0.4 +/- 0.6	5.5 +/- 23.6	0.3 +/- 0.3
	2019	39.0 +/- 122.7	0.7 +/- 1.1	66.6 +/- 238.8	0.4 +/- 1.2	3.8 +/- 5.2	0.3 +/- 0.2	1.6 +/- 2.4	0.3 +/- 0.3
Time of year	Jan-Mar	15.1 +/- 78.4	0.5 +/- 0.9	4.6 +/- 7.5	0.2 +/- 0.2	1.9 +/- 1.4	0.2 +/- 0.2	1.0 +/- 0.8	0.4 +/- 0.3
	Apr-Jun	70.6 +/- 163.5	1.1 +/- 1.6	180.5 +/- 399.3	0.4 +/- 1.3	11.1 +/- 25.4	0.3 +/- 0.2	8.1 +/- 27.6	0.3 +/- 0.2
	Jul-Sep	60.2 +/- 141.5	0.8 +/- 1.3	107.6 +/- 287.8	0.6 +/- 1.9	5.5 +/- 6.6	0.3 +/- 0.2	1.6 +/- 2.4	0.3 +/- 0.2
	Oct-Dec	10.9 +/- 41.9	0.4 +/- 0.3	2.4 +/- 2.4	0.3 +/- 0.4	0.3 +/- 4.0	0.3 +/- 0.4	1.8 +/- 2.8	0.4 +/- 0.3
Religion	Hindu	40.8 +/- 122.4	0.7 +/- 1.2	83.5 +/- 271.5	0.4 +/- 1.4	5.4 +/- 13.9	0.3 +/- 0.4	2.6 +/- 13.8	0.3 +/- 0.3
	Muslim	55.7 +/- 146.3	0.8 +/- 1.3	87.7 +/- 280.8	0.2 +/- 0.1	7.2 +/- 17.5	0.2 +/- 0.2	7.1 +/- 19.9	0.4 +/- 0.4
	Others	0.4 +/- 0.4	0.8 +/- 0.4	1.5 +/- 0.8	0.2 +/- 0.1	2.3 +/- 2.3	0.1 +/- 0.2	1.6 +/- 2.5	0.3 +/- 0.3
Parity	Primigravida	23.0 +/- 98.4	0.5 +/- 0.4	10.1 +/- 36.8	0.2 +/- 0.2	2.6 +/- 4.2	0.2 +/- 0.2	1.4 +/- 1.9	0.4 +/- 0.3
	Multiparous^	48.6 +/- 132.4	0.8 +/- 1.3	106.3 +/- 306.9	0.5 +/- 1.4	6.7 +/- 16.4	0.3 +/- 0.4	4.0 +/- 17.3	0.3 +/- 0.3
BOH	Present	69.7 +/- 153.7	1.0 +/- 1.6	166.1 +/-385.7	0.6 +/- 1.8	11.7 +/- 24.9	0.3 +/- 0.4	8.1 +/- 27.0	0.3 +/- 0.3
	Absent	30.5 +/- 109.3	0.6 +/- 0.9	46.4 +/- 191.9	0.3 +/- 0.9	3.2 +/- 4.4	0.3 +/- 0.2	1.4 +/- 2.2	0.3 +/- 0.2

## Discussion

The prevalence of ToRCH infections varies significantly from region to region, depending upon various factors like climatic conditions, socio-economic status, including personal hygiene, cultural beliefs, dietary habits, and other anthropogenic factors [5,7]. Estimating regional seroprevalence of ToRCH agents from time to time is of immense help in formulating strategies for antenatal screening and guiding physicians in making screening decisions. It is even more important in countries like India that lack national screening programs for ToRCH infections in pregnant women. This prompted us to conduct the present study in the hilly state of Uttarakhand.

Our study observed a TG seroprevalence of 41.2%, among which 13.3% showed anti-TG IgM antibodies indicating active infection posing a threat to the developing fetus. The global prevalence of latent toxoplasmosis in pregnant women was estimated at 33.8%, with a significant association with countries with low income and low human development indices [11]. Singh and Pandit reported an overall Toxoplasma seroprevalence of 45%, which conforms with our finding. Another study from northeast India also reported a high TG seroprevalence of 48% [12]. However, a national serological survey of TG prevalence in India reported a lower seroprevalence of 9.4-19.7% in the north Indian population [13]. Similarly, another study from north India reported seropositivity of 21% in the general population [14]. This comparatively higher TG prevalence in the high-risk group of pregnant women prompts the need for antenatal screening and treatment. In the absence of vaccination against toxoplasmosis, antenatal monitoring and health education of the targeted population (pregnant women) regarding the handling of animals and avoiding consumption of undercooked meat products and raw fruits/vegetables can go a long way in reducing the burden of toxoplasmosis. Poor fetal outcomes and congenital anomalies can be avoided by prevention, timely detection, and treatment. Countries like France, Sweden, and Austria have been successful in reducing the *Toxoplasma gondii* disease burden in humans with the implementation of gestational screening, educational programs, frequent re-testing, and rapid treatment [6,7,15].

In our study, the highest seroprevalence was seen in RV (80%); however, IgM was present only in 3% of cases. This may be due to the extensive rubella vaccination program since 2014 [16]. World Health Organisation, South-East Asia Region had set a 2023 (from earlier 2020) target to eliminate rubella and reduce the burden of congenital rubella syndrome (CRS) [17] by introducing rubella-containing vaccines (RCV) into the public-sector childhood immunization program. Similarly, a study from north India reported the presence of anti-RV antibodies in 82.6% of cases post-vaccination [18]. Many other studies reported a very high prevalence of rubella-specific-IgG in pregnant women [2,4,19-22]. Although some authors question the utility of routine screening of RV in pregnancy [23], it may be instrumental in finding the immunologically naïve (seronegative) pregnant women who are susceptible to RV infection and advising them for preventive measures and vaccination (post pregnancy) for safety in a future pregnancy.

The seroprevalence of CMV in India was reported to be around 80-95% [2,19-21]. Comparatively, we observed less seropositivity of 61.8%. Lachmann et al. reported a seroprevalence of 62.3% in women, which conforms with our finding [24]. Moreover, Hoehl et al. observed a significant decline in seroprevalence between 1988-1997 and 1998-2008 [25]. Changes in lifestyle, such as a well-documented tendency toward smaller households, with fewer young children as possible sources of infection, could be the possible reason for the decline [25]. Proper prenatal/antenatal counseling of pregnant women about personal hygiene, hand washing, reducing exposure to human body fluids (blood, saliva, urine, genital tract secretions, etc.), especially of young children, can further reduce the burden of CMV infection [26].

In the present study, the prevalence of HSV 1 and 2 infection in pregnant women was 42.4%. High seroprevalence of 57-64% for HSV in India was reported by few studies [2,19,27,28]. But in developed nations, HSV seroprevalence is estimated to be around 7-22% [26]. Since the majority (>80%) of neonatal herpes is transmitted during delivery, elective cesarean section and prophylactic acyclovir to infected mothers are recommended to control neonatal herpes [1,19].

It is generally observed that ToRCH seroprevalence increases with increasing age, and this has been well documented [2,14,15,29]. Here we observed a significant increase in TG infection rate with increasing maternal age. The prevalence of RV, CMV, and HSV was also higher in older age groups, but they were not statistically significant (Table 3). Maximum seropositivity of RV was seen in the drier and windy months of January-March, while TG was maximum in the warmer months of April-June. Few studies reported lower seroprevalence of HSV among married Muslim women compared with other religions [29,30]. Contrarily, we observed a significantly higher HSV seroprevalence in pregnant Muslim women. However, this finding might be confounded by other socio-economic factors. The meta-analysis by Van Howe observed that HSV infections are not impacted significantly by circumcision status, while intact men were found to be at a lower overall risk of any sexually transmitted infections (STIs) [31].

Primary infections caused by ToRCH pathogens are the major cause of BOH, and this is well corroborated in published studies both locally [2,19,23] and elsewhere [1,5,22]. This study also observed a higher seroprevalence of ToRCH infections in pregnant women with BOH. The association was strongest with TG, followed by HSV and RV. But the association with CMV was not statistically significant (p-value=0.097) (Table 3). Multiparous women were also at higher risk of having TG, RV, and CMV infection compared with primiparous women, which may be due to increasing maternal age and exposure to ToRCH pathogens in the previous pregnancies. Thus, targeted public health awareness and screening right from the first pregnancy can help in reducing the disease burden of ToRCH infections.

A limitation of the study is that the patients were tested only once. Following up on the patients throughout the pregnancy or further could have revealed important information regarding seroconversion and predictors of infection. Moreover, a multi-centric study could have given a better insight into the variability of ToRCH seroprevalence in different regions of India and better generalization. Another limitation was that we could not gather the actual data of rubella vaccination status, which could have differentiated between protected and naïve populations.

## Conclusions

To conclude, estimating the serological status by assessing the IgM and IgG antibody levels in pregnant women gives an insight into the disease burden of ToRCH infections in this high-risk population responsible for serious fetal consequences. The findings of this study have provided baseline epidemiological data on the seroprevalence of ToRCH infections from this hilly state of Uttarakhand for future in-depth studies. We found an increased seroprevalence of TG and other infections with increasing age, the drier and windy months of January-March favoring RV and warmer months of April-June favoring TG infections, higher prevalence of HSV infections in Muslim pregnant women, and predisposing role of multiparity and BOH towards ToRCH infections. These observations call for an integrated approach of antenatal screening, preventive approaches (like vaccination for RV, consumption of properly cooked food for TG, personal hygiene for CMV, HSV, etc.), and public awareness about transmission and risk factors for controlling ToRCH-related fetal complications.
